# Impact of Controlling a Nutritional Status Score on Wound Healing in Patients with Chronic Limb-Threatening Ischemia after Endovascular Treatment

**DOI:** 10.3390/nu13113710

**Published:** 2021-10-22

**Authors:** Kaori Mine, Makoto Sugihara, Takafumi Fujita, Yuta Kato, Koki Gondo, Tadaaki Arimura, Yosuke Takamiya, Yuhei Shiga, Takashi Kuwano, Shin-ichiro Miura

**Affiliations:** 1 Department of Cardiology, Fukuoka University School of Medicine, 7-45-1 Nanakuma, Jonan-Ku, Fukuoka 814-0180, Japan; kaoringo1110appleappile@gmail.com (K.M.); bcfit85142@gmail.com (T.F.); qqps556d@woody.ocn.ne.jp (Y.K.); tagosa98823@yahoo.co.jp (K.G.); kirinnwine@yahoo.co.jp (T.A.); ytakamiya2007@yahoo.co.jp (Y.T.); yuheis@fukuoka-u.ac.jp (Y.S.); tkuwano1977@gmail.com (T.K.); 2 Division of Cardiology, Fukuoka University Nishijin Hospital, Fukuoka 814-8522, Japan

**Keywords:** chronic limb-threatening ischemia, controlling nutritional status, clinical frailty, delayed wound healing

## Abstract

Background: Chronic limb-threatening ischemia (CLTI) is the most advanced stage of peripheral artery disease. Therefore, a multidisciplinary approach is necessary to avoid major amputation in CLTI patients. Malnutrition worsens the condition of CLTI patients, and therefore, it may be important to evaluate the nutritional status in patients with CLTI. This study was designed to evaluate the baseline patient characteristics and the influence of the controlling nutritional status (CONUT) score on the clinical results. Method and Results: A retrospective, single-center, non-randomized study was conducted to evaluate the associations of death, major amputation, and wound healing rate at 12 months with the CONUT score on admission. Consecutive CLTI patients (mean age 73.2 ± 10.4 years; 84 males) who underwent endovascular therapy (EVT) for infra-popliteal lesions at Fukuoka University Hospital from January 2014 to May 2019 were enrolled and divided into two groups (higher and lower CONUT score groups). The higher CONUT group showed a higher percentage of dialysis (66.7% vs. 33.9%, *p* < 0.001) and a higher clinical frailty scale (5.9 ± 1.4 vs. 4.9 ± 1.9, *p* = 0.005) than the lower CONUT group. Rates of amputation-free survival were 89.5% and 69.8% in the lower and higher CONUT groups, respectively. In addition, rates of wound healing at 12 months were 98.0% and 78.3% in the lower and higher CONUT groups, respectively. Multivariate regression analysis demonstrated that a higher CONUT score was an independent predictor for delayed wound healing (OR: 11.2; 95% CI: 1.29–97.5; *p* = 0.028). Conclusion: An assessment of the nutritional status using the CONUT score could be useful for predicting wound healing, and earlier nutritional intervention may improve the outcome of CLTI patients. Early examination and treatment, along with raising awareness of the issue, may be important for improving the prognosis.

## 1. Introduction

Chronic limb-threatening ischemia (CLTI) is the most advanced stage of peripheral artery disease (PAD). The 2017 guidelines of the European Society of Cardiology (ESC) recommend that revascularization should be attempted whenever possible [1]. Bypass surgery is an effective and durable revascularization strategy, but it is not applicable to all patients with multiple comorbidities and severe frailty. Endovascular therapy (EVT) has become an alternative option for the treatment of CLTI patients. Previous studies have reported a cumulative wound healing rate of 54–86% at one year [2,3]. However, there is a difference between the rate of prevention of major amputation and the rate of wound healing. Even if major amputations are prevented, a certain percentage of patients have ulcers that do not heal. Shiraki et al. reported that nutritional interventions may improve the prognosis of CLTI patients [4]. The Trans-Atlantic Inter-Society Consensus II recommends that factors that adversely affect wound healing, such as heart failure and poor nutritional status, should be assessed and appropriate treatment provided [2]. A recent study has shown that low body mass index (BMI) and serum albumin levels are independent predictors of amputation-free survival (AFS) after EVT in CLTI patients [5]. Malnutrition is known to cause a variety of physical maladies, including skeletal muscle atrophy and decreased immunity. These factors can lead to an immunocompromised state, delayed recovery, and prolonged hospitalization, thus reducing the quality of life (QOL), especially for CLTI patients who require bed rest. The controlled nutritional status (CONUT) score, calculated from the serum albumin, total cholesterol, and the total lymphocyte count, was developed as a simple screening tool to assess nutritional status in hospital populations. Nakagomi et al. reported that poor nutritional status, as assessed by the CONUT score, was significantly associated with a poor prognosis in patients with chronic heart failure [6], and Mizobuchi et al. reported that the CONUT score was associated with adverse clinical events, including all-cause mortality in patients with PAD [7]. These findings suggest that it may be important to screen for nutritional status and may have the potential to improve clinical outcomes and prognosis. However, there have been few reports on the nutritional status as assessed by the CONUT score at admission and the wound healing rate in CLTI patients after EVT. The present study was designed to determine the associations of AFS, delayed wound healing, and major adverse limb events (MALE) with nutritional status at admission in CLTI patients.

## 2. Materials and Methods

### 2.1. Population 

**Figure 1:**
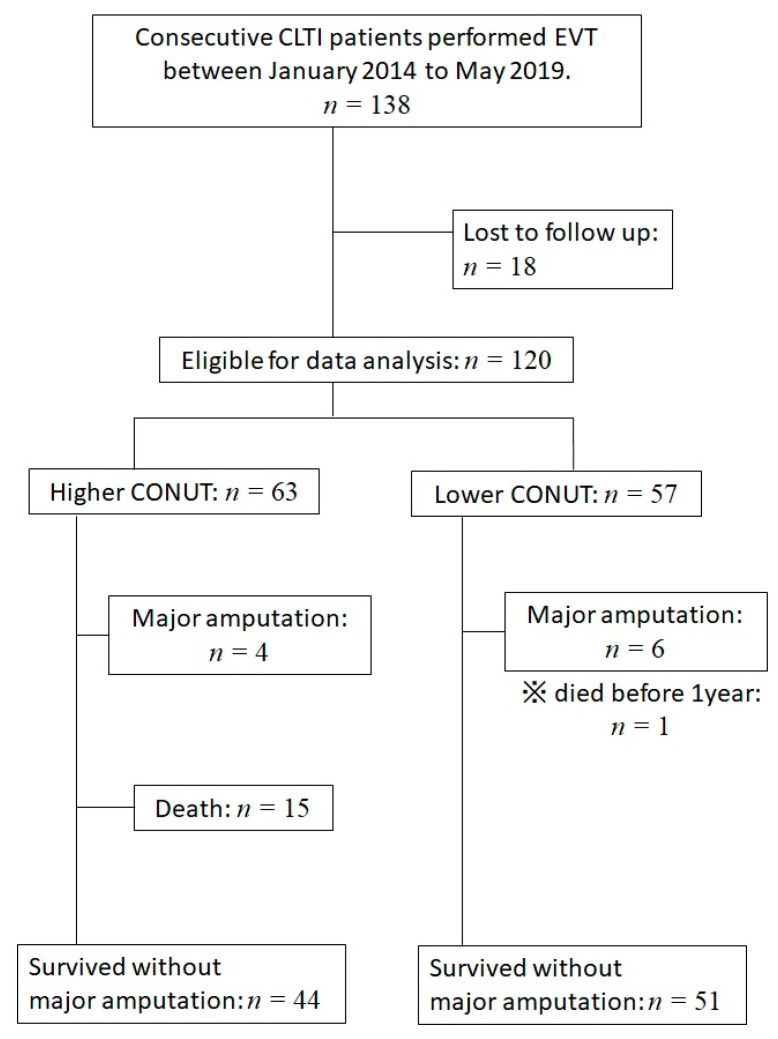
Flowchart of the patient enrollment process. One-hundred thirty-eight patients were enrolled. Eighteen were excluded due to loss of follow up. The analysis was performed on the remaining 120 patients. Abbreviations: CLTI, chronic limb-threatening ischemia; EVT, endovascular therapy; CONUT, controlled nutritional status.

A retrospective, single-center, non-randomized study was conducted to evaluate the associations of death, major amputation, and wound healing rate after 12 months with the CONUT score at the time of EVT. After excluding 18 patients due to loss of follow up, we evaluated the data from 120 consecutive patients (mean age 73.2 ± 10.4 years; 84 males) with CLTI who underwent EVT for infra-popliteal lesions at Fukuoka University Hospital from January 2014 to May 2019 (Figure 1). Patients who underwent primary amputation or primary bypass surgery and patients with acute limb ischemia were excluded at the outset. Demographic characteristics, angiographic profiles, medical history, comorbidities, laboratory data, medications at the time of EVT and long-term prognoses were collected from the patients’ medical records and interviews. The CONUT score was calculated from the serum albumin and total cholesterol levels, and the total lymphocyte count [8]. The score, ranging from 0 to12 points, is categorized as normal (0–1 point), low risk (2–4 points), moderate risk (5–8 points), and severe risk (9–12 points). The serum albumin level is considered to be an indicator of protein stores, the serum total cholesterol level is an indicator of caloric depletion, and the total lymphocyte count is an indicator of decreased immune defense due to malnutrition. The eligible patients were divided into two groups according to their CONUT scores: lower CONUT score group (0–4 points) and higher CONUT score group (5–12 points).

The study protocol was developed in accordance with the Declaration of Helsinki and approved by ethics committee at Fukuoka University (# 2018M087). All patients provided their written informed consent for EVT and the opportunity to opt-out of this study by indicating their preference on the website of Fukuoka University.

### 2.2. Follow-Up

All patients were followed up at 1 month after revascularization and every 3 months thereafter. The follow-up data were collected from the patient’s medical records.

### 2.3. EVT Procedure

The target lesion was evaluated with digital subtraction angiography based on the location of the ulcer. EVT was attempted with the primary goal of creating a single straight line based on the angiosome. However, when angiosome-oriented revascularization was unsuccessful, lesions that were not angiosome-oriented were treated. Successful treatment was defined as when at least one straight line was formed and <25% residual stenosis without any flow reduction was observed in target lesions on the final angiogram. Unsuccessful EVT was defined as failure of the guidewire to pass through the lesion or continued reduction in blood flow after EVT. All patients were given aspirin (100 mg) and/or clopidogrel (75 mg) daily. Patients with above-the-knee lesions and who were treated with stents or a drug-coated balloon received both aspirin and clopidogrel as a rule. The duration of administration of antiplatelet agents was at the discretion of the attending physician. Plastic surgeons made decisions on wound care and amputation during their regular visits. CLTI was defined as chronic ischemic pain at rest, ulceration, or gangrene resulting from objectively proven arterial occlusive disease.

### 2.4. Definitions

Major amputation was defined as an above-ankle amputation. Complete wound healing was defined as the complete epithelialization of all wounds without death or major amputation. Healing time was defined as the time elapsed from the initial EVT to complete epithelialization. Patients who died or underwent major amputation before wound healing was achieved were not counted as delayed wound healing. Delayed wound healing was defined as taking more than 1 year to heal, excluding death or major amputation before complete healing. MALE was defined as the combined event of death, major amputation, or revascularization (surgical or EVT). The Clinical Frailty Scale was used to assess frailty. The Clinical Frailty Scale (CFS) was introduced in the second clinical examination of the Canadian Study of Health and Aging (CSHA) as a way to summarize the overall level of fitness or frailty of an older adult after they had been evaluated by an experienced clinician. This scale ranges from CFS1 (very fit), to 2 (well), 3 (managing well), 4 (vulnerable), 5 (mildly frail), 6 (moderately frail), 7 (severely frail) and 8 (very severely frail) [9]. In addition, patients were divided into a non-to-mild group (CFS 1–5) and moderate-to-severe group (CFS 6–8).

### 2.5. Statistical Analysis

A statistical analysis was performed using Excel 2016 (SSRI, Tokyo, Japan) and EZR ver. 1.41 (Saitama Medical Center, Jichi Medical University), which is a graphical user interface for R (The R Foundation for Statistical Computing, Vienna, Austria, ver.3.5.2). More precisely, it is a modified version of R commander designed to add statistical functions frequently used in biostatistics. Continuous variables are shown as the mean ± standard deviation and were compared between the groups by a t-test. Categorical variables were compared between the groups by a chi-square analysis or Fisher exact test. A Kaplan–Meier analysis (log-rank test) was applied to verify the time-dependent occurrence of clinical outcomes in the lower and higher CONUT groups. A receiver-operating characteristic (ROC) curve analysis was used to determine the cut-off levels of the CONUT score to distinguish between the presence and absence of MALE at the highest possible sensitivity and specificity levels. Baseline characteristics of atherosclerotic risk factors, and the patient’s status including CONUT score and CFS were prescreened using a univariate logistic regression analysis and a multivariate logistic regression analysis was performed to identify independent predictors of AFS and delayed wound healing. The results are presented as the odds ratios (ORs) and 95% confidence intervals (CIs). A value of *p* < 0.05 was considered significant.

## 3. Results

### 3.1. Baseline Characteristics 

**Figure 2:**
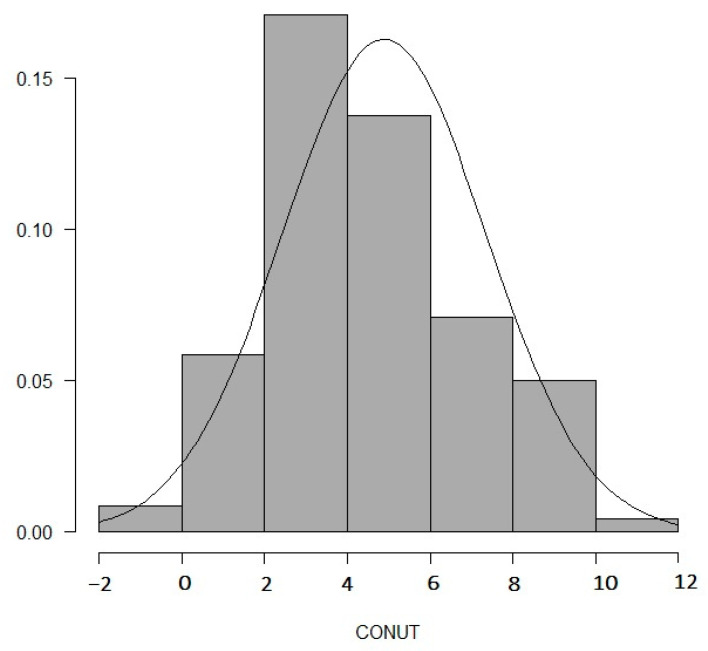
The distribution and the normality of controlled nutritional status (CONUT) scores were evaluated.

The patients were divided into higher (*n* = 63) and lower (*n* = 57) CONUT groups. The CONUT score distribution is shown in Figure 2. The mean value for the entire population was 4.9 ± 2.5. As shown in Table 1, the mean age was 73.2 ± 10.4 years and all 84 (70%) patients were male. There were no significant differences in age, BMI, percentages of hypertension, dyslipidemia, diabetes mellitus, chronic kidney disease, prior PCI or smoking history between the higher and lower CONUT groups. Sixty-two (51.2%) patients in the present study had regular HD. The higher CONUT group had higher % dialysis (*p* < 0.001), lower % Prior CVD (*p* = 0.02) and higher CFS (*p* = 0.005) than the lower CONUT group. There were no significant differences in medications between the two groups at the time of EVT. As shown in Table 2, the higher CONUT group showed lower hemoglobin (*p* < 0.001), low-density lipoprotein cholesterol (LDL-C) levels (*p* < 0.001) and higher CRP (*p* = 0.002) compared to the lower CONUT group. Regarding the components of the CONUT score, there were significant differences in albumin levels (*p* < 0.001), total lymphocyte counts (*p* < 0.001) and total cholesterol levels (*p* < 0.001), which were CONUT score endpoints in addition to albumin levels.

### 3.2. Clinical Outcomes at 12 Months

As shown in Table 3, there was a significant difference in % death at 12 months between the higher and lower CONUT groups (*p* < 0.001). The incidence of MALE in the higher CONUT group (46.0%) was significantly higher than that in the lower CONUT group (22.8%) (*p* < 0.012).

### 3.3. Kaplan–Meier Curves for AFS and Wound Healing in the Higher and Lower CONUT Groups

**Figure 3:**
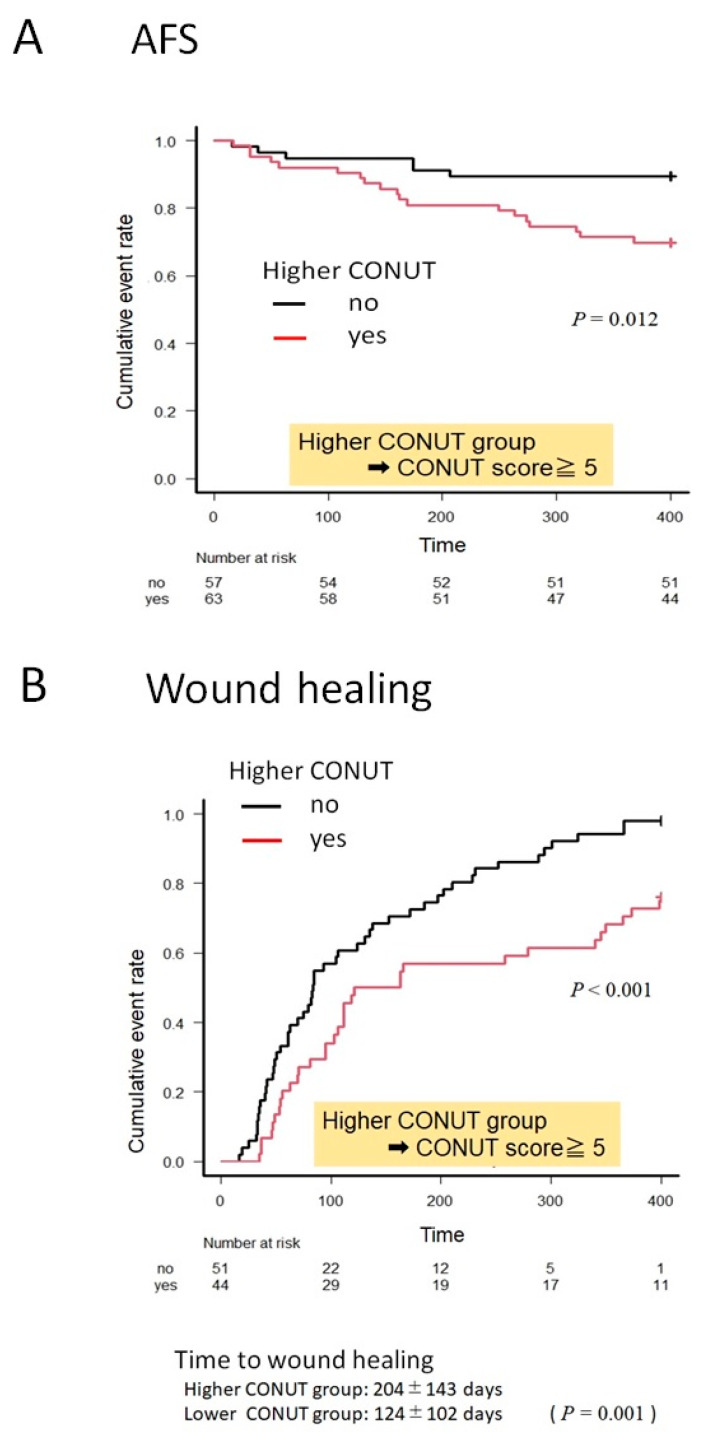
Kaplan–Meier curves for amputation-free survival (AFS) (**A**) and wound healing (**B**) in the higher and lower controlling nutritional status (CONUT) groups.

Figure 3 shows Kaplan–Meier curves for AFS and wound healing in the higher and lower CONUT groups. Time to wound healing was 204 ± 143 days in the higher CONUT group and 124 ± 102 days in the lower CONUT group. The curves demonstrated significant differences in AFS and the wound healing rate between the two groups. In the lower and higher CONUT groups, AFS at 12 months was 89.5% and 69.8%, respectively, and the wound healing rate at 12 months was 98.0% and 77.3%, in the lower and higher CONUT groups, respectively. 

### 3.4. ROC Curve Analysis to Determine the Cut-Off Levels of the CONUT Score to Distinguish between the Presence and Absence of MALE

**Figure 4:**
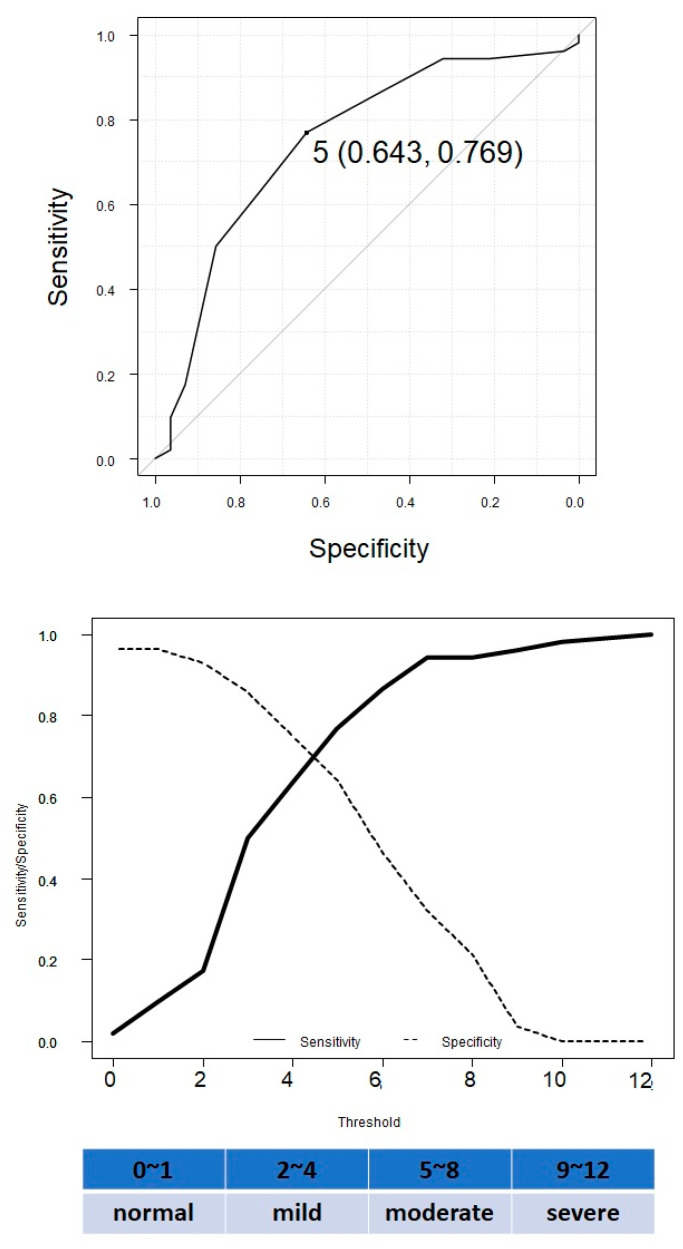
A receiver-operating characteristic (ROC) curve analysis to determine the cut-off levels of the controlling nutritional status (CONUT) score to distinguish between the presence and absence of major adverse limb event (MALE).

We performed a ROC curve analysis to determine the cut-off levels of the CONUT score to distinguish between the presence and absence of MALE at the highest possible sensitivity and specificity levels (Figure 4). The area under the curve (AUC) of the ROC curve was 0.76 and a CONUT score of 5 was considered to be a good threshold indicator. The sensitivity and specificity were 0.769 and 0.643, respectively.

### 3.5. Predictors for AFS and Delayed Wound Healing

A logistic regression analysis to evaluate the association between AFS and delayed wound healing at 12 months is shown in Table 4. A univariate regression analysis demonstrated that higher CONUT (*p* = 0.011) and moderate-to-severe frailty (*p* = 0.002) were significantly associated with AFS. A multivariate regression analysis demonstrated that moderate-to-severe frailty was a predictor of AFS (Hazard ratio, 20.5; 95% confidence interval, 2.61–161.0, *p* = 0.005). A univariate regression analysis demonstrated that CRP > 3 mg/dL (*p* = 0.023) and a higher CONUT (*p* = 0.008) were significantly associated with delayed wound healing. A multivariate regression analysis demonstrated that a higher CONUT score was an independent predictor of delayed wound healing (Hazard ratio, 11.2; 95% confidence interval, 1.29–97.5, *p* = 0.028).

## 4. Discussion

The main finding of this study is that the CONUT score is a useful predictor of delayed wound healing in CLTI patients. The median duration of wound healing after EVT was longer in the higher CONUT group (i.e., worse nutritional status). 

Current clinical guidelines, such as those from The European Society for Clinical Nutrition and Metabolism and the American Society for Parenteral and Enteral Nutrition, recommend that nutritional status be screened during hospitalization and that nutritional support be provided if there is a risk of malnutrition [10,11]. Recent studies have demonstrated the importance of systematic screening, followed by nutritional assessment, and the introduction of individualized nutritional support for patients regardless of their disease status [4,12]. Nutrition screening is the first step in identifying patients with nutritional disorders, especially those who are at risk of malnutrition or suspected to be at risk of malnutrition, and a variety of tools have been developed for this purpose. The next step after screening is a more detailed assessment of nutritional status, which is called “nutritional assessment.” The CONUT score is a nutritional assessment tool that is calculated from the serum albumin levels, total cholesterol levels, and the total lymphocyte count, which can be easily obtained by blood tests [8]. The CONUT score can effectively predict the prognosis and can be calculated from simple parameters. Previous studies have demonstrated that the lymphocyte count and neutrophil/lymphocyte ratio can predict mortality or amputation in CLTI patients [13,14], and that patients with controlled lipid profiles, such as low TC and triglyceride levels in malnourished patients, have poorer outcomes [15,16]. The serum albumin level has been reported to be a predictor of the prognosis in CLTI patients. Recent studies have shown that serum albumin <3 g/dL is strongly associated with delayed wound healing; Azuma et al. reported that albumin <3 g/dL was a negative predictor of wound healing after bypass surgery [17]; Shiraki et al. reported that low albumin is an independent predictor of delayed wound healing after EVT in CLTI patients. [18]. This score may reflect both the nutritional status and inflammation in patients with atherosclerosis. As a result, the CONUT score successfully assessed wound healing rate in CLTI patients in this study. However, there are some points to be noted. When assessed with the CONUT score, lymphocytes and total cholesterol are affected by inflammation and medications such as statins, while albumin levels are susceptible to fluid retention in patients with heart failure. 

Next, we examined the use of the CONUT score as an index for examining MALE. As shown by the ROC curve, a CONUT score of 5 is considered to be a good threshold indicator; a CONUT score of 5 or higher is defined as moderate or severe malnutrition, and therefore, CONUT score is considered to be a good indicator for screening the nutritional status of CLTI patients. Revascularization is an integral part of the treatment of patients with CLTI but is not sufficient by itself. Multidisciplinary treatment, including revascularization, pharmacotherapy, nutritional intervention, and rehabilitation can improve clinical outcomes for CLTI patients. Regarding pharmacotherapy, in the present study, the baseline LDL-C level of CLTI patients was 80 mg/dL. Therefore, the prescription rate of statins in CLTI patients may have been low. 

We analyzed the factors associated with AFS and delayed wound healing and found that moderate-to-severe frailty and malnutrition were independent predictors, respectively. It has been reported that frailty-related factors (such as gait status) are strongly associated with a poor prognosis in CLTI patients undergoing revascularization [5,19,20]. AFS was predicted by moderate-to-severe frailty, and a high CONUT score was not a factor in the present study. Mortality was also significantly higher in the higher CONUT group. These results indicated that severe frailty had a significant impact on life expectancy. Conversely, in patients who survived without major amputation, nutritional status was found to be the most important factor affecting wound healing. Iida et al. reported that CRP > 3 mg/dL predicted major amputation and death [20]. On the other hand, CRP > 3 mg/dL was a predictor of delayed wound healing only in the univariate analysis in the present study. Patients with delayed wound healing may be trapped in a vicious cycle of delayed wound healing due to malnutrition and chronic inflammation. Wound healing and inflammation relief can also help to restore nutritional status. It has been reported that malnutrition and sarcopenia often exist in parallel in individuals due to common factors such as aging, nutrient intake, weight, muscle mass, and decreased physical function [21,22]. Although the efficacy of nutritional therapy for patients with CLTI remains unclear, we found that malnutrition is a risk factor for worse clinical outcomes. We suggest that nutritional therapy may be a new therapeutic target to improve the prognosis of CLTI patients.

## 5. Study Limitations

This study was a single-center study with a small sample size and limited observation period, and there is a possibility of a selection bias for patients who underwent EVT. In addition, the CONUT score was assessed only at the time of admission, and the prognostic impact of changes in the score during the observation period was not evaluated.

## 6. Conclusions

Assessment of the nutritional status using the CONUT score could be useful for predicting wound healing, and earlier nutritional intervention may improve the outcome of CLTI patients. Early examination treatment, as well as raising awareness, are important for improving the prognosis.

## Figures and Tables

**Table 1:** Baseline patient characteristics.

Variables	Overall (*n* = 120)	Higher CONUT (*n* = 63)	Lower CONUT (*n* = 57)	*p*
Patient				
Age, years	73.2 ± 10.4	73.1 ± 9.9	73.3 ± 10.9	0.886
Male sex	84 (70)	41 (65.1)	43 (75.4)	0.237
Body mass index, kg/m^2^	21.6 ± 3.7	21.3 ± 3.5	22.0 ± 3.9	0.280
Hypertension	92 (76.7)	48 (76.2)	44 (77.2)	1.000
Dyslipidemia	65 (54.2)	32 (50.8)	33 (57.9)	0.468
Diabetes mellitus	101 (84.2)	53 (84.1)	48 (84.2)	1.000
Chronic kidney disease	86 (71.2)	47 (74.6)	39 (68.4)	0.544
Hemodialysis	62 (51.2)	42 (66.7)	20 (35.1)	<0.001
Prior PCI	69 (57.5)	39 (61.9)	30 (52.6)	0.357
Prior CVD	21 (17.5)	6 (9.5)	15 (26.3)	0.018
Smoking history	79 (65.8)	44 (69.8)	35 (61.4)	0.343
Chronic hepatitis	1 (0.8)	0 (0)	1 (1.8)	0.475
Ratherford 4/5/6	4 (3.3)/87 (72.5) /29 (24.1)	2 (3.2)/46 (73.0) /16 (25.4)	2 (3.5)/42 (73.7) /13 (22.8)	
Isolated BK lesion	65 (54.1)	35 (55.6)	30 (52.6)	0.871
CONUT score	4.9 ± 2.5	6.8 ± 1.7	2.9 ± 1.1	<0.001
WIfI High-risk	73 (60.8)	43 (68.3)	30 (52.6)	0.086
CFS	5.4 ± 3.7	5.9 ± 1.4	4.9 ± 1.9	0.005
Aspirin	70 (58.3)	39 (61.9)	31 (54.4)	0.355
Thienopyridine	78 (65.0)	42 (66.7)	36 (63.2)	0.706
Cilostazol	13 (10.8)	2 (3.2)	11 (19.3)	0.006
DOAC	9 (7.5)	6 (9.5)	3 (5.2)	0.496
ARB/ACEI	75 (62.5)	32 (50.8)	43 (75.4)	0.007
Statin	73 (60.8)	32 (50.8)	41 (71.9)	0.024
Insulin	19 (15.8)	10 (15.9)	9 (15.8)	0.812

Continuous data are presented as the means ± standard deviations. Categorical data are given as the number (percentage; %). Abbreviations: CONUT, controlled nutritional status; PCI, percutaneous coronary intervention; CVD, cerebrovascular disease; BK. Below the knee; WIfI, Wound, Ischemia, and foot Infection; CFS, Clinical Frailty Scale; DOAC, direct oral anticoagulants; ARB/ACEI, angiotensin receptor blocker/angiotensin-converting enzyme inhibitor.

**Table 2:** Biochemical data.

Variables	Overall (*n* = 120)	Higher CONUT (*n* = 63)	Lower CONUT (*n* = 57)	*p*
Serum albumin (mg/dL)	3.3 ± 0.6	3.0 ± 0.4	3.7 ± 0.4	<0.001
Total cholesterol (mg/dL)	145.1 ± 33.3	135.6 ± 30.8	155.8 ± 33.1	<0.001
Lymphocyte count (10^3^/mL)	1169.4 ± 557.6	968.9 ± 405.1	1394.9 ± 619.8	<0.001
Hemoglobin (g/dL)	11.2 ± 1.8	10.5 ± 1.7	11.9 ± 1.7	<0.001
CRP (mg/dL)	2.5 ± 4.2	3.6 ± 5.2	1.4 ± 1.8	0.002
Total bilirubin (mg/dL)	0.5 ± 0.3	0.5 ± 0.26	0.6 ± 0.4	0.193
HbA1c (%)	6.4 ± 1.1	6.3 ± 1.1	6.5 ± 1.1	0.299
HDL-C (mg/dL)	40.9 ± 13.4	39.4 ± 12.0	42.7 ± 14.7	0.174
LDL-C (mg/dL)	80.7 ± 28.4	72.6 ± 25.5	89.9 ± 29.0	<0.001
TG (mg/dL)	103.2 ± 55.0	98.4 ± 45.3	108.7 ± 64.3	0.311

Continuous data are presented as the means ± standard deviations. Categorical data are given as the number (percentage; %). Abbreviations: CONUT, controlled nutritional status; CRP, C reactive protein; HbA1c, hemoglobin A1c; HDL-C, high-density lipoprotein cholesterol; LDL-C, low-density lipoprotein cholesterol; TG, triglyceride.

**Table 3:** Clinical outcomes at 12 months.

Variables	Overall (*n* = 120)	Higher CONUT (*n* = 63)	Lower CONUT (*n* = 57)	*p*
Death (%)	16 (13.3)	15 (23.8)	1 (1.8)	<0.001
TLR (%)	25 (20.8)	16 (25.4)	9 (15.8)	0.261
MI (%)	1 (0.8)	1 (1.6)	0 (0)	1.000
CVA (%)	4 (3.3)	3 (4.8)	1 (1.8)	0.621
Major amputation (%)	10 (8.3)	4 (6.3)	6 (10.5)	0.515
Minor amputation (%)	47 (39.2)	27 (42.9)	20 (35.1)	0.455
MALE (%)	42 (35.0)	29 (46.0)	13 (22.8)	0.012

Continuous data are presented as the means ± standard deviations. Categorical data are given as the number (percentage; %). Abbreviations: CONUT, controlled nutritional status; TLR, target lesion revascularization; MI, myocardial infarction; CVA, cerebrovascular attack; MALE, major adverse limb event.

**Table 4:** Univariate and multivariate logistic regression analyses of AFS and delayed wound healing.

Predictors of AFS				
	Univariate	*p*	Multivariate	*p*
Male sex	0.44 (0.18–1.13)	0.090		
Diabetes mellitus	0.96 (0.30–3.23)	0.980		
Chronic kidney disease	1.02 (0.38–2.72)	0.967		
Hemodialysis	1.53 (0.63–3.75)	0.351	1.29 (0.45–3.71)	0.640
Anemia (<Hb 9 g/dL)	0.56 (0.22–1.42)	0.221		
CRP (>3 mg/dL)	1.74 (0.70–4.37)	0.236	1.08 (0.39–3.01)	0.885
Higher CONUT	3.67 (1.35–10.0)	0.011	2.24 (0.69–7.23)	0.179
WIfI High-risk	2.02 (0.73–5.56)	0.175		
Moderate-to-severe frailty	23.50 (3.05–181.0)	0.002	20.50 (2.61–161.0)	0.005
**Predictors of delayed wound healing**				
	Univariate	*p*	Multivariate	*p*
Male sex	1.29 (0.32–5.10)	0.340		
Diabetes mellitus	0.37 (0.10–1.37	0.136		
Chronic kidney disease	1.29 (0.33–5.10)	0.716		
Hemodialysis	2.47 (0.71–8.63)	0.157	1.84 (0.45–7.48)	0.396
Anemia (<Hb 9 g/dL)	2.08 (0.63–6.87)	0.232		
CRP (>3 mg/dL)	4.05 (1.22–13.5)	0.023	3.05 (0.81–11.5)	0.099
Higher CONUT	16.70 (2.07–124.0)	0.008	11.20 (1.29–97.5)	0.028
WIfI High-risk	0.57 (0.18–1.83)	0.342		
Moderate-to-severe frailty	1.60 (0.49–5.28)	0.441	0.95 (0.25–3.58)	0.941

Abbreviations: AFS, amputation-free survival; CRP, C-reactive protein; CONUT, controlled nutritional status; WIfI, Wound, Ischemia, and foot Infection. Data are presented as the odds ratio (95% confidence interval), *p* value.

## Data Availability

The data presented in this study are available on request from the corresponding author.
